# The effect of exercise on vascular health in chronic kidney disease: a systematic review and meta-analysis of randomized controlled trials

**DOI:** 10.1152/ajprenal.00152.2023

**Published:** 2023-09-21

**Authors:** Mark D. Davies, Felicity Hughes, Aamer Sandoo, Abdulfattah Alejmi, Jamie Hugo Macdonald

**Affiliations:** ^1^Institute for Applied Human Physiology, School of Psychology and Sport Science, Bangor University, Bangor, Gwynedd, United Kingdom; ^2^Department of Emergency Medicine, Ysbyty Gwynedd Hospital, Bangor, Gwynedd, United Kingdom; ^3^School of Psychology and Sport Science, Department of Sport Science, Bangor University, Bangor, Gwynedd, United Kingdom; ^4^Renal Department, Ysbyty Gwynedd Hospital, Bangor, Gwynedd, United Kingdom

**Keywords:** arterial stiffness, chronic kidney disease, endothelial, exercise

## Abstract

Patients with chronic kidney disease (CKD) are at increased risk of cardiovascular disease. This increased risk cannot be fully explained by traditional risk factors such as hypertension. Endothelial dysfunction and arterial stiffness have been suggested as factors that explain some of the increased risk and are independently associated with important cardiovascular outcomes in patients with CKD. Studies in other disease populations have shown the positive effects of exercise on vascular dysfunction. The aim of this review was to determine whether exercise training interventions improve measures of vascular function and morphology in patients across the spectrum of CKD and which exercise training interventions are most efficacious. A systematic search of Medline, Embase, and the Cochrane Central Register identified 25 randomized controlled trials. Only randomized control trials using an exercise intervention with a nonexercising control group and at least one measure of vascular function or morphology were included. Participants were patients with nondialysis CKD or transplant patients or those requiring dialysis therapy. A systematic review was conducted according to Preferred Reporting Items for Systematic Reviews and Meta-Analyses (PRISMA) guidelines. A meta-analysis was completed for pulse wave velocity, augmentation index, and measures of endothelium-dependent vasodilation. Data from 25 studies with 872 participants showed that exercise training reduced pulse wave velocity and augmentation index but had no effect on endothelium-dependent vasodilation. Subgroup analyses suggested that exercise interventions of at least moderate intensity were more likely to be effective. Limitations included the absence of observational studies or other interventions aimed at increasing habitual physical activity. Further studies are warranted to investigate which are the most effective exercise interventions.

**NEW & NOTEWORTHY** A thorough systematic review and meta-analysis of the effects of exercise training on measures of vascular function in patients with chronic kidney disease, including arterial stiffness and endothelial function, were conducted. Subgroup analyses investigated how differences in exercise training, according to frequency, intensity, type, and timing, have an impact on the efficacy of the intervention.

## Graphical abstract

**Figure:**
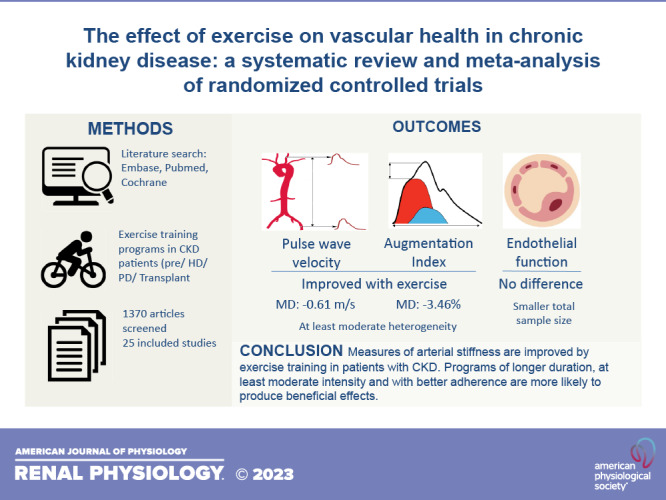

## INTRODUCTION

Cardiovascular disease (CVD) is the leading cause of mortality in patients with chronic kidney disease (CKD) (1). Indeed, CKD is an independent risk factor for CVD with an increased risk of death, cardiovascular events, and hospitalization as the estimated glomerular filtration rate (eGFR) falls (2). Coronary heart disease risk is estimated to be 40% higher in people with reduced kidney function (eGFR < 60 mL/min/1.73 m^2^) (3). The association between CKD and CVD remains even after adjustment for nonmodifiable risk factors (age and sex) and for known vascular disease risk factors (hypertension, diabetes mellitus, and dyslipidemia) as well as adjustment for established vascular disease (coronary heart disease, ischemic stroke, transient ischemic attack, and peripheral arterial disease) (1). A number of “nontraditional” risk factors, which may explain this increased risk, have been suggested and studied. These include uremic bone disease, oxidative stress, and inflammation. Impaired vascular function, i.e., endothelial dysfunction and arterial stiffness, has emerged as a significant factor in explaining some of the excess CVD risk (4, 5), both per se and as a pathway through which other risk factors have their negative consequences.

Endothelial function and arterial stiffness are impaired in patients with CKD. Large artery endothelium-dependent vasodilation is impaired in nondialysis CKD (ND-CKD) (6–8), may decline with falling eGFR (9), and is worse in patients requiring hemodialysis (HD) (7, 8). Similarly, microvascular endothelial function is impaired in CKD (10–12) and end-stage kidney disease (13). Likewise, arterial stiffness is increased across the spectrum of CKD (14–18). Both these aspects of vascular function are associated with important clinical outcomes in patients with CKD. In observational studies, microvascular and large vessel endothelial dysfunction predict cerebrovascular and cardiovascular events in patients with CKD (19–22) and are associated with future eGFR decline in patients with hypertension (23). Measures of arterial stiffness [pulse wave velocity (PWV) and augmentation index (AIx)] have been shown to predict the progression to end-stage kidney disease (14, 24) in ND-CKD and the development of heart failure and other cardiovascular outcomes in both ND-CKD (25, 26) and patients established on HD (27). Thus these measures of vascular function are valuable surrogate markers of cardiovascular outcomes.

Therapeutic options for improving vascular health in patients with CKD are limited. Treatments targeting CKD-associated mineral bone disease have had mixed results, with trials of the phosphate binders sevelamer and lanthanum carbonate showing no benefit on PWV (28, 29); in contrast, a study that examined the effects of vitamin D supplementation on flow-mediated dilation (FMD), a measure of endothelium-dependent vasodilation, demonstrated positive results (30). A study in patients treated with atorvastatin demonstrated a nonstatistically significant slowing of the rate of increase in aortic stiffness (31). Blood pressure treatment is almost ubiquitous in patients with CKD: labetalol has been shown to reduce PWV, although not independently of the reduction in blood pressure (32); treatment with lisinopril and irbesartan had no effect (32, 33). Despite some positive effects in studies and the common use of these treatments in patients with CKD, the need to reduce CVD and vascular dysfunction remains.

Exercise has been considered a potential therapeutic option to target vascular dysfunction in patients with CKD. The physiological effects of exercise, such as increased sheer stress leading to increased nitric oxide (NO) bioavailability, and other vascular adaptations have been extensively reviewed elsewhere (34). Exercise training has been shown to be beneficial in other disease conditions and in health: studies have demonstrated improvements in outcomes, including endothelium-dependent vasodilation, in patients with coronary artery disease (35, 36), chronic heart failure (37), type 2 diabetes mellitus (38), and hypertension (39) and in young patients with prehypertension (40) as well as in healthy men (41). Similarly, exercise training improves PWV in patients with obesity (42), hypertension (43, 44), and established coronary heart disease (45) as well as in healthy postmenopausal women (46).

Considering the importance of this field and the potential for impact, the effect of exercise training on the vascular health of patients with CKD warrants exploration. Large randomized controlled trials of exercise training on vascular health are challenging in patients with CKD; currently, only smaller trials have been completed. One recently published review reported a significant effect of exercise training on measures of arterial stiffness in patients with CKD (47). There was, however, significant heterogeneity in the results, which was not fully explored (only the duration of intervention was considered). Additionally, a global assessment of vascular health using other peripheral measures of vascular function and morphology was not included. Therefore, the aim of this report was to complete a systematic review and meta-analysis to determine the effect of exercise on vascular function and morphology in patients with CKD, using the most up-to-date tools and best practice for statistical analysis and bias assessment. Furthermore, to identify the most effective exercise training programs, the meta-analysis aimed to complete subgroup analyses based on frequency, intensity, time, and type (FITT) principles and to determine the effect in different CKD populations [ND-CKD, HD, peritoneal dialysis (PD), and those with kidney transplant (Tx)].

## METHODS

This systematic review was conducted and reported according to Preferred Reporting Items for Systematic Reviews and Meta-Analyses (PRISMA) guidelines (48). The review was registered with PROSPERO (CRD42021232523).

### Eligibility Criteria

Eligibility criteria were defined a priori. Inclusion criteria were based on participant, intervention, comparison, outcomes, and study design (PICOS) principles. Participants in both exercise and control groups were patients with CKD (nondialysis dependent, HD, PD, or Tx) aged 18 yr or older; the intervention of interest was trials of exercise training at least 4 wk in duration. The comparison group must have been equivalent CKD participants, either completing no exercise (i.e., normal routine care) or completing an attention control intervention (i.e., comparator exercise of a lower intensity). Studies that also included a nonexercise, nonpharmacological intervention, alongside an exercise training intervention, were included as long as it was also provided to the control group. The outcome measures of interest were relevant vascular measures, i.e., assessment of large artery or microvessel function; these include peripheral measures of endothelium-dependent vasodilation (FMD, laser-Doppler imaging or flowmetry and reactive hyperemia index measured by peripheral arterial tonometry) or measures of arterial stiffness or compliance. Also included were measures of consequent structural changes, i.e., carotid intima-media thickness. The study designs of interest were randomized trials. The following studies and publications were specifically excluded: observational studies of habitual physical activity, exercise interventions combined with other intervention (if not provided to the control group), animal experiments, conference proceedings, systematic reviews and meta-analyses, and communication articles or letters to the editor and research protocols. Studies of exercise with outcomes related to arteriovenous fistula maturation (e.g., vessel diameter or peak blood flow) were not included. The language was limited to English.

### Literature Search

A comprehensive search for peer-reviewed manuscripts published in English was performed by two authors (M. D. Davies and F. Hughes) using the following three databases: Medline (1946–present), Embase (1974–present), and the Cochrane Library (1992–present). The date of the last search was December 31, 2022. Search terms included Medical Subject Headings (MeSH) terms and keywords related to CKD, Tx, and dialysis modalities, exercise and physical training terms, and terms related to measures of vascular function or the endothelium. Details are provided in the Supplemental Material. The references of previously published systematic reviews of related questions and of the included articles were also screened.

Titles and abstracts were reviewed by two reviewers (M. D. Davies and F. Hughes). The full text of any trial that was considered potentially relevant by one or both reviewers was then independently assessed against inclusion and exclusion criteria. Any disagreement about the inclusion of a study was decided by a third reviewer (J. H. Macdonald).

### Data Extraction

Data extraction of the selected studies was performed by one reviewer (M. D. Davies). A second reviewer (F. Hughes) checked the extracted data for accuracy. A tailored extraction form was used to collect study characteristics (see Supplemental Material). The authors of the studies were contacted if necessary to gather additional information. The quality of the studies was assessed using the revised Cochrane risk of bias tool for randomized trials (RoB2) (49). This considers potential bias in five domains: bias arising from the randomization process, bias due to deviations from intended interventions, bias due to missing outcome data, bias in the measurement of the outcome, and bias in the selection of the reported result. The overall risk of bias was determined using the criteria as specified in the *Cochrane Handbook* (50): low risk of bias, some concerns, or high risk of bias. Two reviewers (M. D. Davies and F. Hughes) assessed the trials independently and resolved any disagreements through discussion with input from a third reviewer (J. H. Macdonald) if required.

### Statistical Analysis

Data were used from each study’s followup at the completion of the intervention. A random effects model was used for this analysis because of the differences in exercise interventions (duration, intensity, modes of exercise, etc.) used in the included studies. The restricted maximum likelihood estimator (51) was used to calculate the heterogeneity variance τ^2^. Knapp-Hartung adjustments (52) were used to calculate the confidence interval (CI) around the pooled effect. In subgroup analyses with only two studies in a group, where Knapp-Hartung adjustments may be inappropriate (53), the DerSimmonian-Laird CI was also calculated; this is commented on in the results. Where possible, the result at the end of the intervention period, as opposed to the difference from baseline, was used in the meta-analysis and authors were approached to provide these data if not presented in the study report. Outcomes reported as medians and interquartile ranges were converted to means and SDs using the method previously described by Luo et al. (54); CIs and SEs were converted to SDs using the method recommended by the *Cochrane Handbook* (55).

For PWV, where the outcome is on a meaningful and comparable scale (m/s), and for AIx, where the outcome is consistently reported as a percentage of pulse pressure with clinically meaningful relationships between differences in percentages (56), the effect size of each study was calculated as the raw mean difference (MD) between groups. For the endothelium-dependent vasodilation outcomes, which were a combination of different measures, standardized MD (SMD) was calculated using Hedge’s *g* (57).

Heterogeneity was assessed by a variety of measures: the *P* value of *Q* was calculated (testing the null hypothesis that all studies shared a common effect size), as was τ (an estimate of the SD of the true effect size). *I*^2^ was also calculated (an estimate of the proportion of observed variance that is due to real differences in effect size); *I*^2^ values of 25%, 50%, and 75% were considered as low, moderate, and high, respectively (58). The value of τ was used to calculate a prediction interval, a measure of the dispersion of effect sizes, i.e., the range of possible effects that would be found if similar studies were conducted in the future.

Publication bias was assessed by visually inspecting a funnel plot, and by applying Thompson’s regression test, which accounts for residual heterogeneity (59).

Prespecified subgroup analyses included splitting by patient population and by FITT elements of the exercise training prescription. Adherence to the exercise program was used in place of prescribed frequency and time, as the prescriptions were similar across studies. Categories of intensity were assigned based on the description given in the studies. For type, consideration was given to the mode of exercise: intradialytic, aerobic, combined, and walking; these categories were defined post hoc. Finally, the duration of the exercise intervention was considered by subgroup analysis and meta-regression.

Sensitivity analyses were completed by outlier identification and influence analysis (60), including leave-one-out analysis. Subgroup analysis included splitting by patient population and the duration of the intervention.

Risk of bias was assessed by Cochrane RoB2 (49).

All analyses were completed in R [v4.1.1 (61)] in Rstudio [v2022.12.0 (62)] using the meta package [v5.2-0 (63)].

## RESULTS

### Literature Search

**Figure 1.**
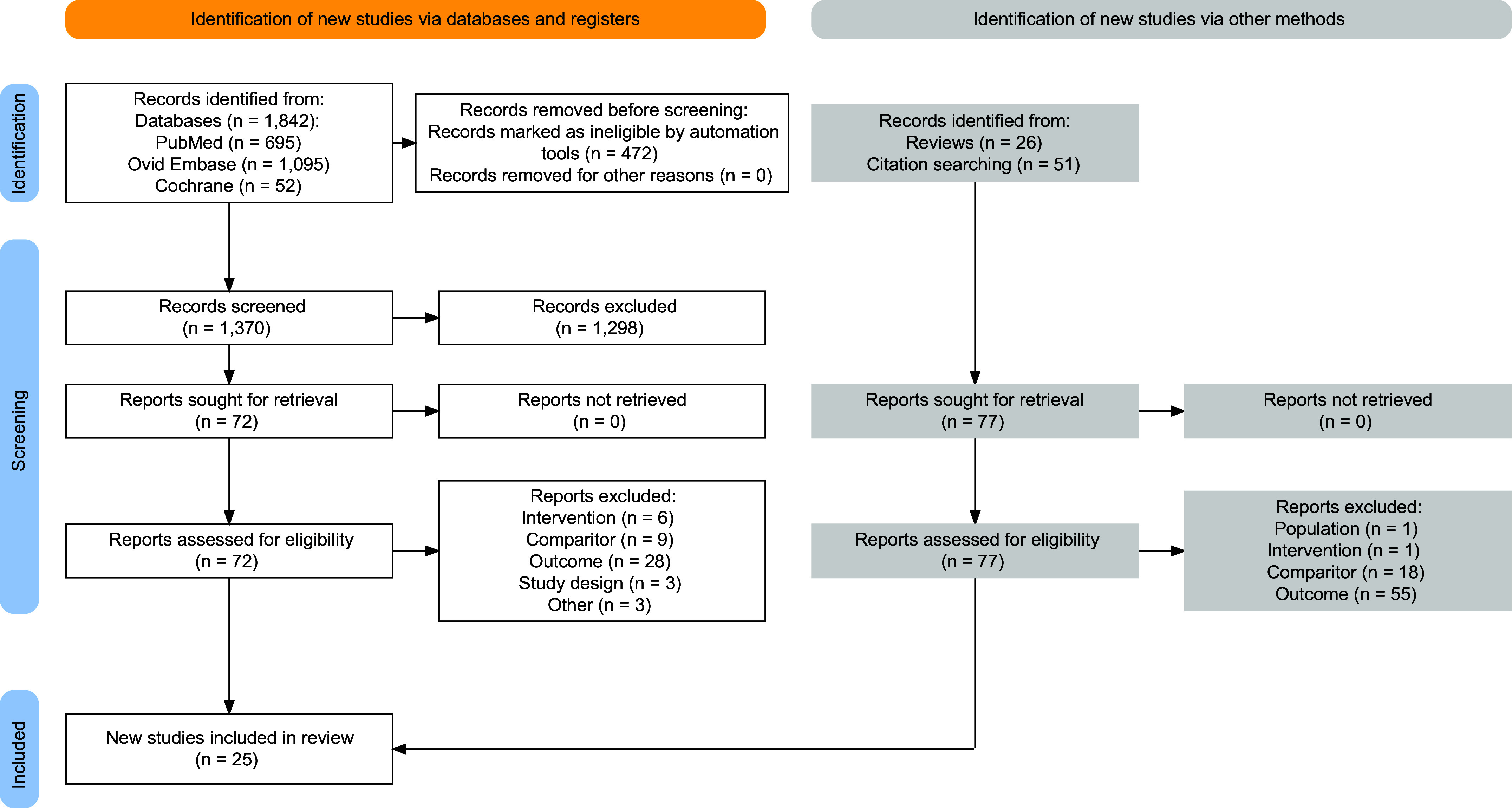
PRISMA 2020-compliant flow diagram using the PRISMA2020 R package (64). Automation tools were used to remove non-English language studies and conference abstracts in the Embase search. In reasons for exclusion, “Comparator” includes single-arm studies without a comparator as well as studies with an inappropriate comparator (e.g., comparator did not receive dietary intervention when the intervention arm did), and “Other” includes one article that was a protocol only, a review article, and a study that was an extended followup of an included study. Some studies may have had multiple reasons for exclusion; the reason recorded is in the order of participant, intervention, comparison, outcomes, and study design (PICOS). PRISMA, Preferred Reporting Items for Systematic Reviews and Meta-Analyses.

We retrieved 1,842 articles in searches from the beginning of each database up to December 2022 from Medline, Embase, and Cochrane Central. Automation tools (language or conference abstracts) excluded 472 articles. Of the 1,370 articles examined for eligibility, 1,298 articles were excluded based on the title or abstract. The full texts of the 72 remaining articles were evaluated. Of these, 23 articles fulfilled the inclusion criteria and were included in the review. An additional two studies were identified through citation searching and the references of subject-related reviews, giving a total of 25 studies. The details of the study selection are shown in the PRISMA flow diagram in Fig. 1.

### Study Selection and Characteristics

The details of the included studies are shown in Table 1. In the 25 studies, there was a total of 875 participants; the total numbers of participants in the final followup, and assessed for the outcome of interest, ranged from 18 (79, 86, 89) participants to 156 (69) participants. One study included an additional intervention group, besides the exercise training and usual care groups, of low-frequency electrical muscle stimulation (77); this group was excluded from this review. Greenwood et al. (68) compared aerobic and resistance groups separately and Koh et al. (73) compared intradialytic and home exercise groups separately; these groups are combined in the meta-analysis to avoid counting the control group twice. The control group was usual care in all studies apart from three: one study included whey protein supplementation (71) and another study included dietary intervention (75); both of these, respectively, were also provided to the exercise training group. One other study used stretching and balance exercises in the control group, aiming to keep the heart rate (HR) at <50% HR reserve (84). The exercise training intervention in all studies consisted of aerobic, resistance, or combined exercise. There was a mixture of purely home, unsupervised exercises (74, 76, 80, 83, 87); home after some weeks supervised (75, 85, 88); a combination of supervised in center and home throughout the study (68, 79, 89); those that were supervised or in center throughout, including intradialytic exercise studies (65–67, 69–72, 77, 78, 81, 82, 84, 86); and one that compared intradialytic with home exercise (73). The interventions ranged from 10 to 52 wk in duration; the median duration was 24 wk. Fifteen of the 25 studies were of <6 mo (26 wk) in duration. One study was a crossover study, where groups swapped from exercise training to control, and vice versa, at 3 mo (86); the results from the end of the first 3 mo, before the crossover, were used for meta-analysis.

**Table 1.** Characteristics of included studies including study design, participant population and numbers, and details of the exercise training intervention employed

Study	Study Design	Population	Setting/Supervision	Duration, wk	Aerobic Activity	Resistance Activity	Frequency, times/wk	Intensity Prescription	Overall Intensity Description	Target Time, min
Assawasaksakul et al. (65)	RCT, ET vs. UC	HD	Intradialytic: supervised	26	Cycle ergometer	Increased breaking resistance on ergometer	3	Up to RPE 12	Light to moderate	60
Cooke et al. (66)	RCT, ET vs. UC	HD	Intradialytic; supervision not specified	16	Pedaling, RPE 12–16		3	RPE 12–16	Moderate to vigorous	variable
Graham-Brown et al. (67)	RCT, ET vs. UC	HD	Intradialytic: supervised	26	Cycling		3	RPE 12–14	Moderate	30
Greenwood et al. (89)	RCT, ET vs. UC	CKD 3–4	2× supervised; 1× home	52	Recumbent exercise bikes	Upper and lower body; elastic bands, free weights, and weight machines; 80% 1RM	3	80% HRR	Moderate to vigorous	60
Greenwood et al. (68)	RCT, aerobic vs. resistance vs. control	Tx	2× supervise; 1× home.	52	Recumbent stationary exercise cycles/ treadmill/ elliptical trainer	Upper and lower body; 80% 1RM, up to 3 × 10	3	80% HRR; RPE 13–15	Moderate to vigorous	Aerobic: 40; resistance: 60
Greenwood et al. (69)	RCT, ET vs. UC	HD	Intradialytic. Supervised	26	Cycle ergometer	Lower extremity; ankle weights and elastic bands	3	40–75% V̇o_2_ reserve	Moderate to vigorous	40
Headley et al. (70)	RCT, ET vs. UC	CKD 3 (eGFR 30–59)	In center: supervised	16	Variety of apparatus		3	50–60% V̇o_2peak_	Moderate	55
Jeong et al. (71)	RCT, ET, and whey protein vs. whey protein vs. UC	HD	Intradialytic: supervised	52	Cycle ergometer		3	RPE 12–14	Moderate	45
Kirkman et al. (72)	RCT, ET vs. UC	CKD 3-5	In-center: supervised	12	Cycling, walking/jogging, or elliptical		3	60–85% HRR, RPE 12–16	Moderate to vigorous	45
Koh et al. (73)	RCT, intradialytic vs. home exercise vs. UC	HD	Intradialytic: home: supervised	24	Cycle ergometer/ walking		3	RPE 12–13	Light to moderate	45
Kosmadakis et al. (74)	ET vs. UC	CKD 4–5	Home: no supervision	26	Walking		5	RPE 12–14	Moderate	30
Leehey et al. (75)	RCT, ET and diet vs. diet	CKD 2–4	Home: in-center first 12 wk	52	Treadmill (+/−) elliptical/cycle)	Elastic bands, hand weights, weight machine	3	45–85% V̇o_2peak_	Moderate	60
Manfredini et al. (76)	RCT, ET vs. UC	HD and PD	Home: no supervision	26	Walking at 70–120% walking speed at baseline		3		Light	20
McGregor et al. (77)	RCT, ET vs. EMS vs. UC	HD	Intradialytic: supervised	10	Cycle ergometer			40–60% V̇o_2_ reserve; RPE 12–14	Moderate	60
Mihaescu et al. (78)	Prospective study, ET vs. UC	HD	Intradialytic: supervised	12		Elastic bands, dumbbells, ankle weights. 70–85% 10RM	3	RPE 12–14	Moderate	40
Mustata et al. (79)	RCT, ET vs. UC	CKD 3–4 (eGFR 15–60)	2× supervised in center; 3× home	52	Treadmill, cycle, elliptical, walking (for home exercise)		Up to 5	40–60% V̇o_2peak_; RPE 12–15	Moderate	60
NCT03197038 (80)	RCT, ET vs. UC	CKD 3–4	Home: no supervision	24	Walking		3		Moderate	60
Oliveira E Silva et al. (81)	RCT, ET vs. UC	HD	Intradialytic: supervision not specified	16	Cycle ergometer		3	65–75% HR_max_	Moderate	45
Riess et al. (82)	RCT, ET vs. UC	Tx	In center: supervised	12	Cycle ergometer and treadmill	Lower extremity; 50% 1RM; 2 × 15	Aerobic: 3; resistance: 2	60–80% V̇o_2peak_; RPE 11–13	Moderate	30–60
Sheshahdri et al. (83)	RCT, ET vs. UC	HD and PD	Home: no supervision	12	Walking (with pedometers); instructed to increase step count by 10% each week				Light	
Sprick et al. (84)	RCT, ET vs. stretching	CKD 3–4	In center: supervised	12	Stationary bicycle		3	60–85% HRR	Moderate to vigorous	45
Thompson et al. (85)	RCT, ET vs. UC	CKD 3–4	Home: in center once per week for first 8 wk	24	Treadmill, cycling, elliptical, walking	Isometric exercises	3	40–59% HRR; RPE 3–4	Moderate	
Toussaint et al. (86)	Randomized cross-over, ET vs. UC	HD	Intradialytic: no supervision	12	cycle ergometer. no set intensity		3		Not set	30
Uchiyama et al. (87)	RCT, ET vs. UC	PD	Home: no supervision	12	Walking	Elastic bands upper and lower body exercises; 70% 1 RM; 1 × 10 repetitions	Aerobic: 3; resistance: 2	40–60% HR_peak_; RPE 11–13	Light to moderate	30
Van Craenenbroeck et al. (88)	RCT, ET vs. UC	CKD 3–4	Home: in center for first 4 wk	12	Cycle ergometer (intermittent: 4 × 10 min)		7	90% HR at AT	Moderate	40

Participant numbers are the total included in final analysis for each study. AT, aerobic threshold; CKD, nondialysis chronic kidney disease; ET, exercise training; HD, hemodialysis; HRR, heart rate reserve; HR_peak_, heart rate peak; HR_max_, maximum heart rate; V̇o_2peak_, peak oxygen consumption; PD, peritoneal dialysis; RCT, randomized controlled trial; RM, repetition maximum; RPE, rating of perceived exertion; Tx, kidney transplant; UC, usual care.

### Modality and Intensity of Exercise Training Interventions

All 25 studies included aerobic training as part of the intervention, apart from Greenwood et al. (89), which had an aerobic-only arm and a resistance-only arm, and Mihaescu et al. (78), which was a resistance-only intervention. Of the 23 others that used an aerobic element, 8 studies also included resistance training (65, 68, 69, 75, 82, 85, 87, 89).

Walking, cycling (stationary bikes or recumbent pedaling), treadmills, or elliptical machines were used for the aerobic components of the exercise interventions. These were prescribed with a range of intensities. Five of the studies were of light or light to moderate intensity: these included two walking interventions that prescribed walking by step count or percentage of baseline speed (76, 83), another walking intervention with a set rating of perceived exertion (RPE) of 11–13 (or 40–60% peak HR), and two intradialytic interventions that prescribed an RPE of up to 12 and 12–13 on the Borg 6–20 scale (65, 73).

Aerobic interventions were of moderate intensity in 13 studies. Five studies used peak oxygen consumption, ranging from 40 to 80% V̇o_2peak_ in four studies (70, 75, 79, 82) or 40 to 60% V̇o_2_ reserve (77). Three moderate studies used HR to prescribe intensity: 40–59% of HR reserve (85), 65–75% maximum HR (81), and 90% of HR at anaerobic threshold (88). Four used RPE alone, at 12–14 (67, 71, 74, 78). One walking intervention described itself as moderate, but no details are available of how this was prescribed (80).

Six studies were of moderate to vigorous intensity, although only one of these was described as such in the report (72). The set intensities included the percentage of HR reserve, from 60% to 85% (68, 72, 84, 89) and 40 to 75% V̇o_2_ reserve (69). The remainder used RPE alone, at 12–16 (66).

Most studies that used relative measures of V̇o_2_ or HR also used RPE as an indicator or target for intensity (65–67, 71–74, 77–79, 82, 85, 87, 89). One study of intradialytic exercise prescribed no intensity target (86).

Five of the nine studies that included resistance exercises prescribed these as a percentage of 1 or 10 repetition maximum (78, 82, 87, 89). A mixture of elastic bands, free weights, ankle weights, and weight machines was used. Two studies specified lower extremity resistance exercises only (69, 82). One study used isometric exercises (85). One study incorporated a resistance component by increasing breaking resistance on the cycle ergometer during intradialytic exercise (65).

### Risk of Bias

**Figure 2.**
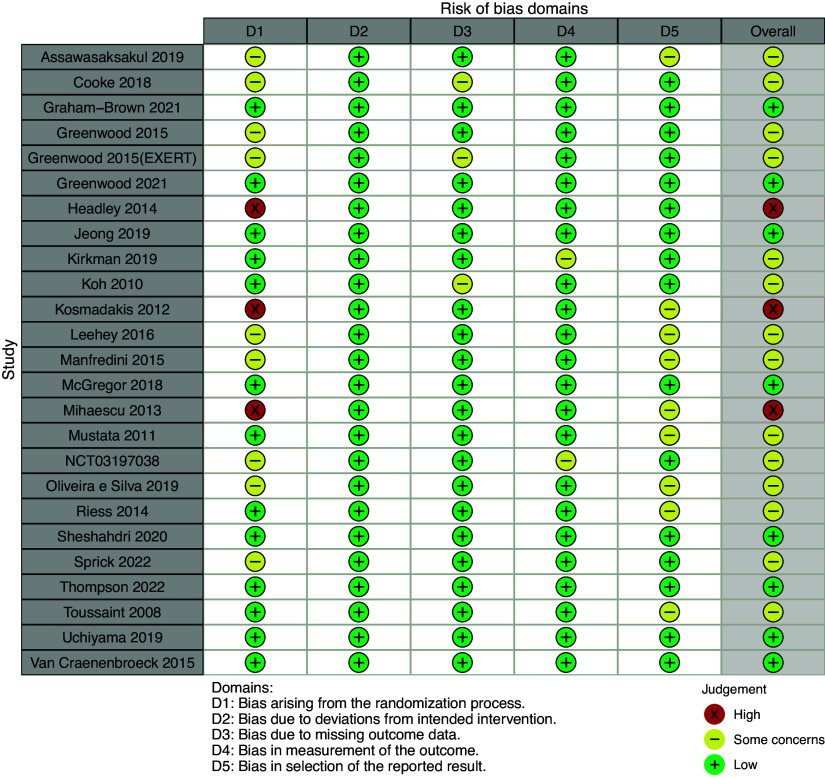
RoB2 traffic light plot for the risk of bias of all included studies. This was produced using the R package robvis (90).

The risk of bias was assessed for all included studies (Fig. 2). The majority of studies, 14 of 25 studies, had “some concerns” as their overall risk of bias rating; 7 studies were of “low risk” and 3 studies were of “high risk.” Details of the RoB2 assessment and risk of bias bar plots are provided in the Supplemental Material.

#### Bias arising from randomization.

Fourteen of the included studies were of low risk with regard to bias from the randomization process. Two studies (70, 74) were of high risk: the participants were allocated to groups by the order in which they were recruited with no concealment. The remainder (9 of 25 studies) were of some concern as they either provided no information on allocation concealment (66, 68, 75, 81, 84, 89) or provided insufficient information on the randomization process (76, 78, 80).

#### Bias due to deviation from the intended intervention.

Unsurprisingly, given the nature of the intervention, none of the studies blinded participants or those providing the intervention to group assignment. Despite this, all studies were of low risk of bias for *domain 2* as there were no deviations from the intended intervention because of the trial context.

#### Bias due to missing outcome data.

Two of the studies are of some concern due to missing data: both had missing data from participants withdrawn for medical reasons, which were potentially relevant to the outcome of interest: 3 of 15 participants (66) and 4 of 41 participants (73) in the exercise groups.

#### Bias in measurement of the outcome.

Fifteen studies did not blind outcome assessors to group allocation or there was no information on blinding of assessors; it is unlikely that awareness of allocation influenced the assessment, although it is possible in two studies, which measured FMD (72, 80). The remainder of the studies were of low risk.

#### Bias in selection of the reported result.

Nine of the 25 included studies were of some concern for selection of the reported result. In all relevant cases, this was due to a lack of a prespecified analysis plan and/or the outcome (PWV, etc.) was not listed as a prespecified outcome of interest in the study’s clinical trials register entry, e.g., on clinicaltrials.gov.

### Outcome Measures

#### Arterial stiffness.

The main measures of arterial stiffness used in the included studies were assessments of PWV (19 studies) and AIx (11 studies). A variety of measures of PWV were used in the included studies. Carotid-femoral PWV was the most common method of PWV analysis and was used by all but 3 studies reporting PWV: 11 studies measured this using an applanation tonometry technique (66, 70–74, 77, 78, 81, 85, 86, 88) and 5 studies used an oscillometric device (65, 68, 78, 89, 91). One study used MRI to measure aortic PWV (67), and another study measured brachial-ankle PWV by an oscillometric device (87). Details were not available for the other study reporting PWV (80). Two studies (74, 85) measured carotid-radial PWV in addition to carotid-femoral PWV. This is considered to be a measure of peripheral artery stiffness but may also reflect endothelial function (92, 93). In the meta-analysis, the carotid-femoral PWV results were used.

AIx was measured either at the brachial (72, 78), radial (66, 70, 71, 73, 79, 86), or carotid arteries (80, 88) or was unspecified (74, 81). Three studies reported AIx adjusted for HR (AIx75) (66, 70, 71); one study reported both adjusted and unadjusted AIx (73). Where available, adjusted AIx was used in the meta-analysis.

##### Pulse wave velocity.

**Figure 3.**
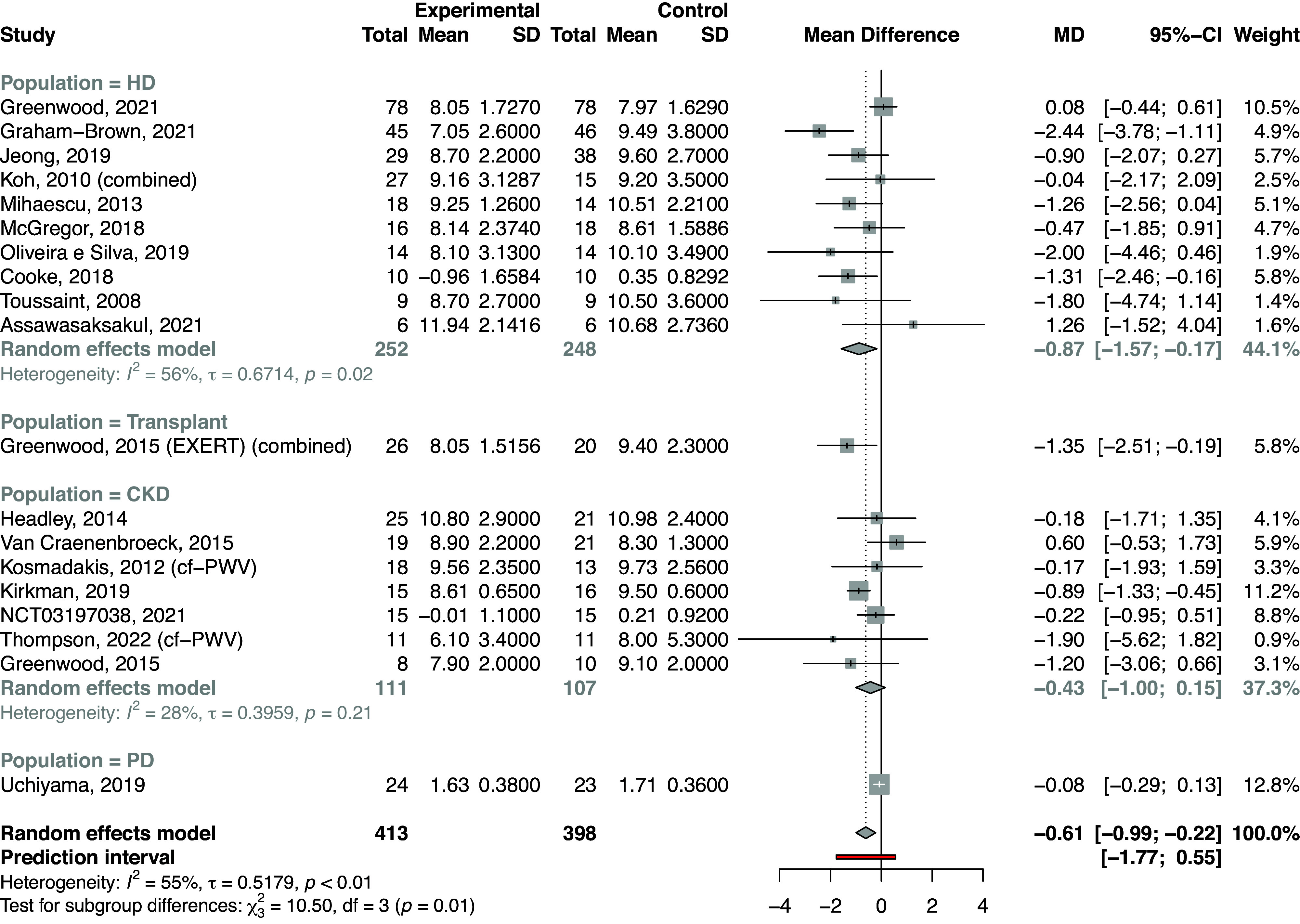
Forest plots of the effect estimates [mean difference (MD)] with 95% confidence intervals (CI) for pulse wave velocity (in m/s) between exercise interventions and control groups for all included studies, with subgroup analysis of the chronic kidney disease (CKD) population. cf-PWV, carotid-femoral PWV; HD, hemodialysis; PD, peritoneal dialysis; *I*^2^, estimate of the proportion of observed variance that is due to real differences in effect size; τ, estimate of the SD of the true effect size.

The results of the meta-analysis for PWV indicate that exercise reduced (i.e., improved) arterial stiffness as measured by PWV (in m/s) [MD = –0.61 m/s, 95% CI: (–0.99, –0.22), *P* < 0.0038; Fig. 3]. Tests for heterogeneity showed a significant *Q* statistic, *P* < 0.01, and relatively high τ (0.52) (*I*^2^ = 55%), suggesting that overall a moderate amount of observed variance was due to real differences in effect sizes. The prediction interval, based on the τ measure of the SD of the effect size, indicates the range of possible effects that would be found if similar studies were conducted in the future. The prediction interval ranged from –1.77 to 0.55 m/s, indicating that, due to the heterogeneity of results, new studies in this field may find marked reductions in PWV, but significant improvement is not guaranteed.

A prespecified subgroup analysis by CKD subpopulation, i.e., hemodialysis (HD), PD, ND-CKD, and Tx, showed significant differences between subgroups (χ^2^ = 10.5, *P* = 0.01). Significant effect sizes (MD) were found in only the HD subgroup [MD = –0.87 m/s (–1.57, –0.17)] and Tx subgroup [MD = –1.35 m/s (–2.51, –0.19)], which had only one study. Even within the HD subgroup, there was significant between-study variance (*P* = 0.02) and moderate real differences in effect sizes (*I*^2^ = 56%). In the ND-CKD subgroup, there was a nonsignificant reduction in PWV [MD = –0.43 m/s (–1.00, 0.15)]; there was no significant heterogeneity (*P* = 0.21, τ = 0.40, *I*^2^ = 28%). There was one study in the PD population with no significant difference in PWV between exercise training and control [MD = –0.08 m/s (–0.29, 0.13)].

In place of frequency and timing of exercise prescription, subgroup analysis explored whether there was an impact of participant attendance at exercise sessions on the effect on PWV, looking at an arbitrary grouping of < or ≥80% of sessions attended. There was a significant effect of exercise training on PWV in the studies where session attendance was ≥80% [MD = –0.66 m/s (–1.27, –0.05)] with low heterogeneity [*P* = 0.23, *I*^2^ = 26%, although still relatively high τ (0.36)] compared with a nonsignificant effect in the studies with <80% attendance [MD = –0.77 (–1.69, 0.15)] with significant, at least moderate, heterogeneity (*P* < 0.01, τ = 0.83, *I*^2^ = 67.5%). The χ^2^ test for subgroup differences, however, was not significant (*P* = 0.81), reflecting the high heterogeneity and wide CI of the <80% attendance subgroup. The results of PWV subgroup analyses results are shown in Table 2.

**Table 2.** Summary of subgroup meta-analyses for pulse wave velocity

Analysis/Subgroups	Number of Studies	MD	95% CI	τ	*I*_2_	Subgroup Differences
Session attendance						
<80%	7	−0.77	[−1.69, 0.15]	0.83	67.5%	*P* = 0.81
80%+	7	−0.66	[−1.27, −0.05]	0.36	25.8%	
Intensity category						
Light to moderate	3	−0.07	[−0.38, 0.23]	0.00	0.0%	*P* < 0.01
Moderate	10	−0.71	[−1.38, −0.03]	0.65	44.3%	
Moderate to vigorous	5	−0.78	[−1.58, 0.02]	0.54	65.3%	
Exercise type						
Combined	4	−0.07	[−1.21, 1.07]	0.28	13.8%	*P* < 0.01
Aerobic	11	−0.88	[−1.45, −0.31]	0.52	32.3%	
Walking	4	−0.09	[−0.18, −0.01]	0.00	0.0%	
Resistance	2	−1.43	[−4.16, 1.30]	0.00	0.0%	
Duration						
3–6 mo	10	−0.62	[−1.35, 0.11]	0.70	51.8%	*P* = 0.05
12 mo	3	−1.14	[−1.77, −0.51]	0.00	0.0%	
<3 mo	6	−0.45	[−1.16, 0.27]	0.49	69.2%	

CI, confidence interval; MD, mean difference; *I*^2^, estimate of the proportion of observed variance that is due to real differences in effect size; τ, estimate of the SD of the true effect size.

An analysis comparing subgroups of prescribed exercise intensity suggested that interventions of at least moderate intensity (e.g., RPE >12) had a greater effect on PWV (*P* value for subgroup differences of <0.01; Supplemental Fig. S4). In the moderate-intensity intervention studies, there was an overall statistically significant MD of –0.71 m/s (–1.38, –0.03) and without significant heterogeneity. While interventions that were moderate to vigorous did not have a significant reduction in PWV [MD = –0.77 m/s (–1.58, 0.02)], there was significant heterogeneity in this subgroup result. The exclusion of Greenwood et al. (69) where there was an attendance of just 47%, resulted in a significant MD in PWV in favor of exercise training [–1.00 m/s (–1.34, –0.66)].

Exercise type was explored by categories of exercise modality. In the subgroup analysis comparing aerobic, resistance, combined (aerobic with a resistance element), and walking interventions, there were significant differences across the subgroups (*P* < 0.01; Table 2 and Supplemental Fig. S5). A significant improvement in PWV was seen in aerobic exercise interventions [MD = –0.88 m/s (–1.45, –0.31)], with moderate heterogeneity (τ = 0.52, although not significant in the *Q* test, *P* = 0.14). Walking interventions affected a very small reduction in PWV [MD = –0.09 m/s (–0.18, –0.01)]. Resistance exercise also produced a marked reduction in PWV [MD –1.43 m/s; with a Knapp-Hartung adjustment to calculate CI, this was not a significant reduction (–4.16, 1.30)]. CI by the DerSimmonian-Laird method was also calculated, as only two studies in the subgroup, τ = 0: this was much narrower (–2.44, –0.42). Combined aerobic and resistance interventions produced no significant effect [MD = –0.07 m/s (–0.75, 0.61)]. A large proportion of the results in the combined subgroup was produced by Greenwood et al. (69), where adherence was low; the remaining studies were small with high variance. An alternative analysis, which created a separate intradialytic cycling category, showed this was the only aerobic subgroup with a significant effect. Given the exploratory nature of this subgroup analysis (the subgroups were chosen post hoc), the results should be interpreted with caution.

Finally, analysis using subgroups of duration of intervention tended toward significance (*P* value for subgroup differences = 0.051). Interventions of 52 wk in duration produced a reduction in PWV [MD = –1.14 m/s (–1.89, –0.38)], with low heterogeneity, but all other groupings did not, with a high heterogeneity of effect (Table 2 and Supplemental Fig. S6). In contrast, meta-regression with duration of intervention (in wk) as a continuous variable did not suggest that duration significantly influenced the studies’ effect sizes (test of moderators *P* = 0.362); a bubble plot is provided in Supplemental Fig. S7. There were no interventions of length between 6 and 12 mo. Taken together, these analyses suggest that exercise interventions of at least 12 mo are likely to produce significant effects, but the effectiveness of interventions shorter than 6 mo is influenced by other factors.

##### Augmentation index.

**Figure 4.**
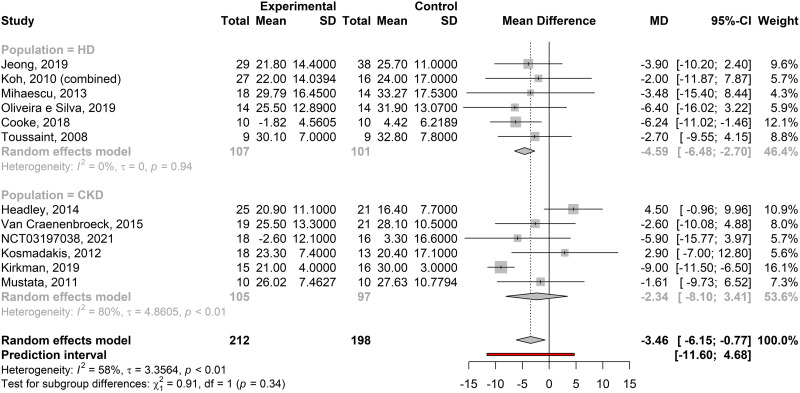
Forest plots of the effect estimates [mean difference (MD)] with 95% confidence intervals (CI) for the augmentation index (in %) between exercise interventions and control groups for all included studies, with subgroup analysis of the chronic kidney disease (CKD) population. HD, hemodialysis; *I*^2^, estimate of the proportion of observed variance that is due to real differences in effect size; τ, estimate of the SD of the true effect size.

Meta-analysis of studies reporting AIx showed a significant MD between exercise training and control groups [MD = –3.46% (–6.15, –0.77), *P* = 0.02] but with significant heterogeneity (*P* = 0.0061, τ = 3.36, *I*^2^ = 58%). As with PWV, the only population subgroup with a significant effect was the HD subgroup, although a χ^2^ test for subgroup differences was not significant (*P* = 0.34; Fig. 4). The prediction interval range of –12.22% to 5.42% indicates that, as with PWV, although future studies may demonstrate marked reductions in AIx with exercise interventions, they may also find small increases.

Subgroup analyses for AIx were performed as for PWV. There were no significant differences between subgroups of attendance percentage, in studies where this was reported, or in analysis of exercise modality or duration of intervention (see Table 3). Exercise intensity subgroup analysis suggested a greater benefit from moderate- to vigorous-intensity exercise compared with light or moderate prescriptions. The significant difference between groups was driven by a very large effect (reduction in AIx) in the moderate to vigorous subgroup. As there were only two studies in that subgroup, by the Knapp-Hartung method, the CI was very wide (Table 3) and overlapped with the other groups, suggesting that the difference between subgroups was not significant [by the DerSimmonian-Laird, CI: (–10.63, –6.17)].

**Table 3.** Summary of results for subgroup analyses of effects of exercise on augmentation index

Analysis/Subgroups	Number of studies	MD	95% CI	τ	*I*_2_	Subgroup Differences
Session attendance						
Session attendance = 80%+	5	−3.06	[−9.44, 3.31]	4.67	81.7%	*P* = 0.58
Session attendance = <80%	3	−4.60	[−11.17, 1.98]	0	0.0%	
Exercise intensity						
Light to moderate	1	−2.00	[−11.87, 7.87]			*P* < 0.01
Moderate	8	−1.40	[−4.87, 2.05]	2.3	10.6%	
Moderate to vigorous	2	−8.40	[−22.86, 6.05]	0.15	0.6%	
Exercise modality						
Exercise type = aerobic	9	−3.45	[−6.83, −0.06]	3.66	67.5%	*P* = 0.95
Exercise type = resistance	1	−3.48	[−15.40, 8.44]			
Exercise type = walking	3	−2.40	[−14.85, 10.05]	0	0.0%	
Duration of intervention						
Duration (mo) = 12 mo	2	−3.04	[−17.14, 11.06]	0	0.0%	*P* = 0.34
Duration (mo) = 3−6 mo	6	−2.03	[−7.40, 3.34]	3.92	53.8%	
Duration (mo) = <3 mo	4	−5.67	[−11.35, 0.02]	2.96	43.9%	

CI, confidence interval; MD, mean difference; *I*^2^, estimate of the proportion of observed variance that is due to real differences in effect size; τ, estimate of the SD of the true effect size.

##### Other measures of arterial stiffness or compliance.

Assawasaksakul et al. (65) used the cardiac ankle vascular index as another measure of aortic stiffness; no differences were found either within or between groups. Riess et al. (82) used small and large artery compliance by diastolic wave form decay; no difference was seen between treatment groups at the end of the intervention period. Finally, Manfredini et al. (76) measured the arterial stiffness index, a measure of arterial stiffness made by oscillometric blood pressure measurements at all four limbs (reported in m/s, the same as PWV). There was a significant increase in the arterial stiffness index in the control group compared with a nonsignificant decrease in the exercise training group; however, there was no between-group difference at the end of the intervention (7.67 m/s with exercise training and 8.7 m/s in control, *P* = 0.09). Measures of carotid-radial PWV in two studies showed no difference between exercise and control groups [MD = 0.44 (–1.13, 2.00) (85) and –0.11 (–1.16, 0.94) (74)].

##### Sensitivity analyses.

Outlier analysis for studies that reported PWV identified one study as a possible outlier, as the CI of that study did not overlap with the CI of the pooled effect (67). Influence analysis suggested that the result of this study was extreme compared with the pooled effect. When this study was removed in a leave-one-out analysis of studies measuring PWV, MD remained significant, in favor of exercise [MD = 0.49 (–0.82, –0.15), *P* < 0.01]. This was the only study to use MRI to measure aortic PWV, which may account for a measurement of greater effect. No outliers were identified in studies that measured AIx. A complete leave-one-out analysis for all studies showed that the effect size on both PWV and AIx remained significant despite the removal of any single study; this included removal of the one study with an overall high risk of bias (74). The removal of certain studies could reduce heterogeneity, as expressed by *I*^2^ (Supplemental Figs. S8 and S9).

#### Endothelial function.

**Figure 5.**
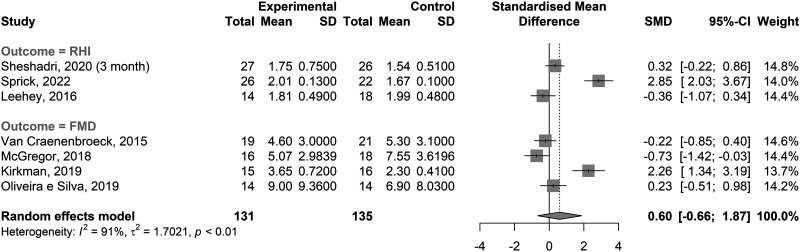
Forest plots of effect estimates [standardized mean difference (SMD)] with 95% confidence intervals (CI) for endothelial function measures between exercise interventions and control groups for all included studies [flow-mediated dilation (FMD) and reactive hyperemia index (RHI) as measured by peripheral arterial tonometry]. All results are end of trial means, not changes from baseline. *I*^2^, estimate of the proportion of observed variance that is due to real differences in effect size; τ, estimate of the SD of the true effect size.

There was only a small number of trials reporting measures of endothelium-dependent dilation: FMD and reactive hyperemia index, measured by peripheral artery tonometry. Therefore, these were considered in a single endothelial function meta-analysis (Fig. 5). No significant difference was shown between exercise training and control groups [SMD = 0.60 (–0.66, 1.87), *P* = 0.29]. Heterogeneity was high (*P* < 0.0001, τ = 1.3047, *I*^2^ = 91.2%). In addition to FMD, Kirkman et al. (72) measured endothelium-dependent vasodilation function using laser-Doppler technique flowmetry. The plateau response to local heating (thermal hyperemia) was significantly improved in the exercise training group compared with no change in the control group (*P* = 0.01).

#### Carotid intima-media thickness.

Carotid intima-media thickness was measured in one study (81); there was no difference in carotid intima-media thickness either within the exercise training group, pre- and postintervention, or between groups.

#### Publication bias.

**Figure 6.**
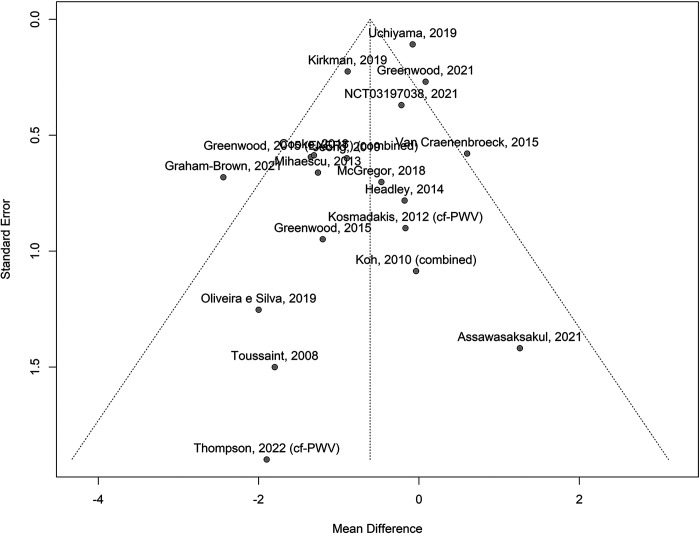
Funnel plot of the mean difference in pulse wave velocity (PWV) among patients with chronic kidney disease after exercise training interventions compared with control. Individual mean differences (in m/s) in PWV in each study are plotted against a measure of each study’s precision (the SE of the mean difference); any asymmetry in the plot indicates the degree of publication bias in the effect estimates. cf-PWV, carotid-femoral PWV.

**Figure 7.**
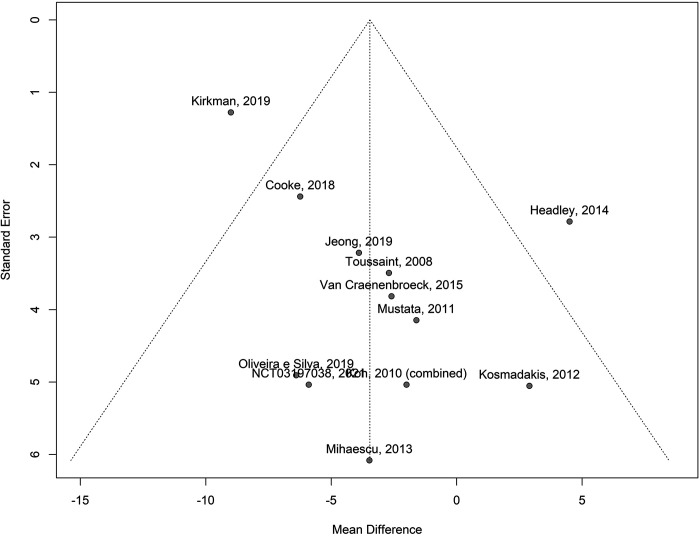
Funnel plot of the mean difference in augmentation index among patients with chronic kidney disease after exercise training interventions compared with control. Individual mean differences (in %) in the augmentation index in each study are plotted against a measure of each study’s precision (the SE of the mean difference); any asymmetry in the plot indicates the degree of publication bias in the effect estimates.

First, visual analysis suggested that the publication bias of studies reporting PWV was unlikely as the individual study results were distributed symmetrically about the mean effect size in each meta-analysis. Second, meaningful publication bias was unlikely as a number of the included studies were of small sample size without significant results. Indeed, inspection of the funnel plot visually (Fig. 6) did show some minor asymmetry but did not suggest a marked “small-study effect,” i.e., overrepresentation of small studies with large effects (94). A linear regression test of funnel plot asymmetry was not significant (*P* = 0.21). Similarly, nonsignificant, minor asymmetry was seen in the funnel plot for studies that reported AIx (*P* = 0.26; Fig. 7).

## DISCUSSION

This systematic review and meta-analysis of the effects of exercise training in patients with CKD on measures of vascular function demonstrated a significant improvement in arterial stiffness as measured by PWV and AIx. No significant effect was found on measures of endothelium-dependent vasodilation as assessed by brachial artery FMD or peripheral artery tonometry.

Reducing arterial stiffness is a very relevant clinical outcome, as elevated arterial stiffness is a risk factor for cardiovascular mortality in patients with ND-CKD and undergoing HD (25, 95) as well as predicting a decline in kidney function in the predialysis CKD population (96, 97). Both PWV and AIx are independently associated with coronary artery disease burden in patients with CKD (98). It has been suggested, based on meta-analysis data in a broad range of populations, that a change in PWV of 0.5 m/s is a clinically meaningful difference (99) (based on a 15% reduction in cardiovascular and all-cause mortality associated with a 1-m/s reduction) (100). Thus the mean reduction found in the present meta-analysis, of 0.61 m/s, likely represents a clinically meaningful difference in PWV and may translate into a reduction in mortality. Based on observational data, a 10% reduction in AIx would translate into a 27–38% reduction in major cardiovascular events (101, 102). The reduction in AIx found in the present analysis was just 3.4%; again, this may translate into a small but relevant effect on CVD.

The results should be interpreted with caution because of the significant heterogeneity in effect sizes, with 55% (PWV) and 58% (AIx) of the heterogeneity probably due to true differences in effect. The prediction intervals for PWV and AIx were –1.8 to +0.6 m/s and –12.2% to +5.4%, respectively. The prediction intervals (based on the τ measure of the SD of the effect size) indicate the range of possible effects that would be found if similar studies were conducted in the future. Due to the heterogeneity of the study findings, the prediction intervals extend down to very meaningful reductions in the measures of arterial stiffness but also cross 0, suggesting that implementation of the exercise training interventions used in these studies does not guarantee beneficial effects. Understanding the reasons for the heterogeneity would help guide future research and implementation.

Overall, exercise improved arterial stiffness in studies in patients with CKD. In subgroup analysis, however, it was only in the HD subgroup that there was a significant effect for both PWV and AIx. There was also a significant effect of exercise on PWV in patients from the Tx subgroup, although this was only measured in one study (68). The reason for the differences in effect between the subgroups of the CKD population is not clear. Further subgroup analyses attempted to explain the heterogeneity of effect. The studies that reported PWV or AIx as outcomes of interest in patients undergoing HD were all supervised, intradialytic interventions. In this situation, the patients involved are somewhat of a “captive audience,” with attendance at sessions being, practically speaking, mandatory, although patients may choose not to engage in the exercise when they attend their dialysis session. Only two intradialytic studies did not find a mean reduction in PWV. One of these two intradialytic studies, the largest study in the HD population, had the lowest reported adherence to exercise, 47% (69). This lends credence to the idea that, on the whole, intradialytic exercise is more effective, at least partially, because of better attendance and, therefore, better compliance with exercise. An analysis by a subgroup of attendance combining all CKD populations showed significant benefits on PWV in the 80%+ group. While the between subgroup difference was not significant, this reflected the residual heterogeneity in the <80% group.

Subgroup analyses by the intensity of the exercise prescribed showed significant between-subgroup differences for both PWV and AIx. These differences suggested that higher intensity interventions, at least moderate for PWV and moderate to vigorous for AIx, were more likely to produce a reduction in arterial stiffness.

Analysis by differing durations of intervention suggested, for PWV, that interventions of 12-mo duration were more likely to produce a significant effect, and the subgroup analysis tended toward statistical significance (*P* = 0.051).

Taken together, these subgroup analyses attempted to overcome the heterogeneity in effect sizes seen in the main analyses for PWV and AIx. While caution should be taken in interpreting the results of multiple comparisons, these subgroup analyses suggest that, probably, more is better, i.e., that interventions are more likely to be effective if they are of longer duration or higher intensity prescription or if participants have higher adherence. The type of exercise appears to be less important in determining the effect.

The effect of exercise training on measures of endothelial function was not significant. Compared with measures of arterial stiffness, relatively few studies assessed endothelial function and there was significant heterogeneity. Subgroup analyses were not performed because multiple comparisons would not be appropriate given the small number of studies. Studies that did have significant positive effects (72, 84) were both of moderate to vigorous intensity and were fully supervised. The few studies that reported both endothelium-dependent vasodilation and arterial stiffness measures showed a similar pattern of effect in both measures, i.e., if aortic stiffness improved, FMD was also improved. Mechanistically, we would expect similar effects with both types of vascular outcome measures. Changes in vasodilatory function and arterial stiffness represent an overlapping stage of atherosclerosis, which is characterized by a reduction in NO bioavailability, increased smooth muscle tone, and breakdown of elastic fibers, along with collagen deposition in the vessel wall (103, 104). In mechanistic studies, measures of arterial stiffness and endothelium-associated vasodilatory function are correlated (105, 106). Furthermore, studies with a variety of interventions often showed similar effects for both arterial stiffness and endothelial function outcomes (107–110). The absence of an effect of exercise on endothelium-dependent vasodilation in patients with CKD in the present analysis, while having a positive effect on arterial stiffness, is unexpected and likely reflects the small number of studies investigating this outcome and the study characteristics.

The present analysis adds to the body of evidence that has demonstrated the positive effects of exercise on measures of vascular health in a variety of healthy and disease populations (35, 37, 38, 41–44, 46).

There are numerous physiological mechanisms whereby exercise may have beneficial effects on vascular function. The main mechanism of action appears to be due to increased laminar shear stress (34, 111). This is a frictional force created by the flow of viscous blood over the endothelial cell surface (112), which is increased by physical exercise, secondary to increased cardiac output and the mechanical action of skeletal muscle contraction and relaxation (113). Shear stress is an important stimulus to the endothelium, having a role in the stimulation of NO production and vascular remodeling (114). Effects of exercise and increased shear stress include increases in NO production and reduced oxidative stress, which improves NO availability (111, 115, 116). Indeed, exercise has been shown to reduce markers of oxidative stress in patients with CKD (117, 118).

Arterial stiffness is impacted not only by enhanced vasodilatory function but also by other mediators of blood vessel tone. Exercise has been shown to reduce resting muscle sympathetic nerve activity and vasoconstrictors, including endothelin-1 and angiotensin II (34, 119), leading to enhanced relaxation of the vessel. In addition to alterations in mediators of blood vessel tone, structural changes to arterial walls contribute to their stiffening, including changes in the balance of elastin and collagen to favor greater deposition of the latter tissue (120). For example, in mouse studies, exercise has resulted in reductions in the levels of collagen types I and III, and it is possible that exercise may also alter structural elements in humans (121). However, structural alterations to the vascular wall occur over a longer time period and are more likely to occur in exercise interventions conducted over a greater number of weeks to months.

A previously published review has also examined the effect of exercise on measures of arterial stiffness (47). Despite some differences in methodology, and additional studies, our findings, of the effect on PWV and AIx, are consistent with Wang et al. (47). That review, however, did not identify the significant heterogeneity that we have explored in this review. The analysis of effects on measures of endothelial function is also new.

There are some limitations to this review and its results. There was some concern of bias in nearly all the studies included in this review, with nearly half having some concerns in multiple domains. The largest single domain with concerns was “bias in measurement of the outcome” due to the absence of blinding of assessors. The degree to which this bias may have affected the results cannot be assessed, but it is a reason for caution in interpreting the data presented here. This review focused on randomized controlled trials of exercise training interventions. While this study design is generally considered to provide the most robust evidence of the effect of an intervention, it is a limitation as it ignores other potentially relevant research, such as observational studies of habitual physical activity. Additionally, other interventions (such as educational programs) aimed at increasing habitual physical activity were not included. Finally, this review only considered measures of vascular function that are assessed by various imaging modalities and not biomarkers, such as levels of NO. These have not been included as they are another step removed from clinically meaningful endpoints, but future reviews may choose to focus on them to provide details on vascular health at the molecular level.

Patients with CKD are known to be sedentary, with reduced levels of physical activity with advancing disease in the predialysis population and reducing further after the commencement of dialysis (122–124). Higher levels of physical activity are associated with improved outcomes in patients with CKD, including reduced cardiovascular mortality in observational studies (125, 126). Increasing physical activity, therefore, is an attractive target to improve the health of patients with CKD. Studies of exercise training, however, in patients with CKD have yet to demonstrate improvements in hard endpoints, such as a survival benefit. Rather than being an absence of effect, this may be due to the short length of followup and the small sample sizes; we are not aware that survival has been measured as an outcome of interest in any exercise training studies in this population. As such, and until such studies are conducted, the measurement of noninvasive surrogate markers, or predictors, of CVD is an important outcome of interest in studies aiming to increase physical activity in patients with CKD. This systematic review and meta-analysis have shown that exercise training in patients across the spectrum of CKD has beneficial effects on measures of arterial stiffness. No effect was found in measures of endothelium-dependent vasodilation, but this analysis was limited by the small number of studies. The positive effect on arterial stiffness adds further impetus to the growing interest in getting patients with CKD active. Further research in this area should focus on understanding the exercise interventions that are most effective in improving vascular function and other measures of cardiovascular risk as well as exploring ways to increase adherence to exercise prescriptions.

## DATA AVAILABILITY

Data will be made available upon reasonable request.

## SUPPLEMENTAL MATERIAL

10.6084/m9.figshare.23272232.v1 Supplemental Details S1: https://doi.org/10.6084/m9.figshare.23272232.v1.

10.6084/m9.figshare.23272223.v2 Supplemental Details S2: https://doi.org/10.6084/m9.figshare.23272223.v2.

10.6084/m9.figshare.24032433.v1 Supplemental Figs. S1–S3: https://doi.org/10.6084/m9.figshare.24032433.v1.

10.6084/m9.figshare.23280107.v1 Supplemental Fig. S4: https://doi.org/10.6084/m9.figshare.23280107.v1.

10.6084/m9.figshare.23272229.v3 Supplemental Fig. S5: https://doi.org/10.6084/m9.figshare.23272229.v3.

10.6084/m9.figshare.23280185.v2 Supplemental Fig. S6: https://doi.org/10.6084/m9.figshare.23280185.v2.

10.6084/m9.figshare.23280230.v2 Supplemental Fig. S7: https://doi.org/10.6084/m9.figshare.23280230.v2.

10.6084/m9.figshare.23280230.v2 Supplemental Figs. S8 and S9: https://doi.org/10.6084/m9.figshare.23280230.v2.

## GRANTS

The PhD research of M.D.D. is supported by Awyr Las (Registered Charity No. 1138976).

## DISCLOSURES

No conflicts of interest, financial or otherwise, are declared by the authors.

## AUTHOR CONTRIBUTIONS

M.D.D., A.S., and J.H.M. conceived and designed research; M.D.D., F.H., and J.H.M. analyzed data; M.D.D. prepared figures; M.D.D. drafted manuscript; M.D.D., F.H., A.S., and J.H.M. edited and revised manuscript; M.D.D., F.H., A.S., A.A., and J.H.M. approved final version of manuscript.
